# MiR-190 ameliorates glucotoxicity-induced dysfunction and apoptosis of pancreatic *β*-cells by inhibiting NOX2-mediated reactive oxygen species production

**DOI:** 10.7717/peerj.13849

**Published:** 2022-08-10

**Authors:** Huinan Lu, Junyu Yang, Juan Li, Huiping Yuan

**Affiliations:** 1 The Key Laboratory of Geriatrics, Beijing Institute of Geriatrics, Institute of Geriatric Medicine, Chinese Academy of Medical Sciences, Beijing Hospital/National Center of Gerontology of National Health Commission, Beijing, P.R. China; 2 Academy for Advanced Interdisciplinary Studies, Peking University, Beijing, China; 3 Peking-Tsinghua Center for Life Sciences, Beijing, China; 4 Department of Biomedical Engineering, College of Engineering, Peking University, Beijing, China

**Keywords:** T2DM, Glucotoxicity, β-cell, miR-190, Cybb, NOX2, ROS

## Abstract

Glucotoxicity-induced pancreatic *β*-cell failure contributes to the development of type 2 diabetes mellitus (T2DM). Accumulating evidence reveals that miRNAs play a critical role in regulating pancreatic *β*-cell function and survival. In this study, we employed a self-assembled cell microarray (SAMcell)-based functional screening assay to identify miRNAs that are capable of regulating the dysfunction of *β*-cells induced by glucotoxicity. Among 62 conserved miRNAs we tested, miR-190 was identified as a candidate regulator that could effectively restore insulin expression in NIT-1 cells under high-glucose (HG) stimulation. Further analyses demonstrated that miR-190 was significantly down-regulated in HG-treated NIT-1 cells, as well as in the pancreas of diabetic mice. Mechanistic studies showed that Cybb is the direct target gene of miR-190, which encodes the gp91phox protein, a subunit of the NOX2 complex. Furthermore, both miR-190 overexpression and Cybb knockdown inhibited apoptosis and improved glucose-stimulated insulin secretion (GSIS) in HG-stimulated NIT-1 cells by attenuating the excessive production of reactive oxygen species (ROS). More importantly, a targeted delivery of mPEG-PCL-g-PDMAEMA nanoparticles/miR-190 complexes (PECgD NPs/miR-190) to the pancreas significantly ameliorated hyperglycemia, decreased fasting serum insulin levels, and improved glucose tolerance in diabetic mice. Taken together, our findings suggest that the miR-190/Cybb axis plays an important role in glucotoxicity-induced pancreatic *β*-cell failure. Restoring miR-190 expression levels may be a possible therapeutic strategy to protect *β*-cells in T2DM.

## Introduction

Diabetes mellitus is a chronic endocrine and metabolic disease, which is characterized by hyperglycemia. According to a report released by the International Diabetes Federation (IDF), in 2021, the global prevalence of diabetes mellitus among 20–79-year-old adults was estimated to be 10.5% (537 million). Diabetes prevalence is expected to climb to 12.2% (783.2 million) by 2045 (Sun et al., 2021). This increasing prevalence of diabetes mellitus worldwide has become a significant burden to healthcare systems.

Over 90% of diabetes cases are type 2 diabetes mellitus (T2DM), which results from pancreatic *β*-cell defects and insulin resistance (Saltiel, 2001). Chronic hyperglycemia-induced pancreatic *β*-cell glucotoxicity is proposed to play a critical role in the pathogenesis of T2DM (Stumvoll, Goldstein & Van Haeften, 2005). Under glucotoxic conditions, elevated plasma glucose leads to pancreatic *β*-cell apoptosis and dysfunction which further exacerbates the disease (Poitout & Robertson, 2008). Accumulating evidence shows that pancreatic *β*-cells exposed to hyperglycemic conditions for a long period of time generate excess reactive oxygen species (ROS), which leads to increased oxidative stress and cellular damage (Robertson, 2004; Yan, 2014). NADPH oxidases of the NOX family are proteins that transfer electrons across biological membranes, and their biological function is to generate ROS (Bedard & Krause, 2007). NOXs overactivation is responsible for *β*-cell failure in diabetes (Nakayama et al., 2005; Oliveira et al., 2003; Uchizono et al., 2006). The phagocyte NADPH oxidase (NOX2) is the first identified NOX family member that is a multi-component system comprising a ph91phox/p22phox-formed membrane-associated catalytic heterodimer as well as cytosolic components (Groemping & Rittinger, 2005). The expression of gp91phox (encoded by the Cybb gene) has been reported to be significantly upregulated in diabetic islets, and NOX2-derived ROS is a major contributor to increased cellular oxidative stress (Nakayama et al., 2005; Syed et al., 2011). In our previous studies, we demonstrated that gp91phox is upregulated in HG-treated NIT-1 cells, and that the suppression of gp91phox substantially restores glucotoxicity-induced impaired insulin synthesis and secretion (Yuan et al., 2010a).

MicroRNAs (miRNAs) are small non-coding RNAs that negatively regulate gene expression at the post-transcriptional level (Krol, Loedige & Filipowicz, 2010). Studies have shown that miRNAs participate in the regulation of various developmental and pathological processes, and their dysregulation is associated with many diseases (Rupaimoole & Slack, 2017), including diabetes (Eliasson & Esguerra, 2020; Guay et al., 2011). To date, a multitude of miRNAs have been discovered to play important roles in *β*-cell failure in diabetes. For instance, miRNAs including miR-34a (Lovis et al., 2008), miR-146 (Lovis et al., 2008), miR-21 (Roggli et al., 2010), miR-29 (Roggli et al., 2012), and miR-200 (Filios et al., 2014) have been shown to be involved in diabetic *β*-cell apoptosis; and miR-9 (Plaisance et al., 2006), miR-24 (Zhu et al., 2013), miR-375 (El Ouaamari et al., 2008), miR-204 (Lovis, Gattesco & Regazzi, 2008) and many more have been identified to be associated with insulin synthesis and secretion. However, a full understanding of how these small, but powerful regulators function during glucotoxicity-induced *β*-cell failure is still unknown.

In this study, we found that the downregulation of miR-190 is an important mediator of glucotoxicity-induced *β*-cell failure, while the overexpression of miR-190 exerts protective effects against glucotoxicity-induced *β*-cell damage by attenuating NOX2-mediated ROS over-generation. Moreover, the mPEG-PCL-g-PDMAEMA nanoparticles (PECgD NPs)-mediated *in vivo* delivery of miR-190 ameliorates diabetic symptoms, indicating that miR-190 may act as a potential therapeutic target for T2DM.

## Materials & Methods

### Cell culture

The NIT-1 mouse pancreatic *β*-cell line (American Type Culture Collection CRL-2055) was cultured with low-glucose (LG) Dulbecco’s modified Eagle’s medium (five mmol/L glucose; Gibco, Waltham, MA, USA) and supplemented with 10% fetal bovine serum (Gibco, Waltham, MA, USA) in incubators at 37 °C with 5% CO2. To establish a glucotoxic pancreatic *β*-cell model, the NIT-1 cells were exposed to 33.3 mmol/L D-glucose (HG) for 48 h as previously described (Yuan et al., 2010a).

### Animals

The 8-week-old, male db/db (leptin receptor-deficient) and ob/ob (leptin-deficient) mice (C57BL/KsJ) used in this study were purchased from the animal center of Peking University. Age-matched male C57BL/6J mice were used as controls. All mice were housed under a 12/12-h light/dark cycle with up to five animals per cage and access to food and water ad libitum. All procedures involving experimental animals were performed in accordance with protocols approved by the Institutional Animal Care and Use Committee of Peking University (No. COE-XiJZ-1) and conformed to the Guide for the Care and Use of Laboratory Animals (NIH publication No. 86-23, revised 1985). In this study, all efforts were made to minimize the suffering of the animals, and certain criteria for excluding and euthanizing the mice prior to the planned end of the experiment were established as follows: any mouse that lost 20% or more body weight, showed a lack of appetite, demonstrated weakness or inability to obtain food or water, appeared to be dying, or had an infection would be excluded from the study and euthanized by cervical dislocation.

### Immunofluorescence

Cells were washed with ice-cold phosphate-buffered saline (PBS) and fixed with 4% paraformaldehyde in PBS for 20 min at room temperature. After being washed three times with PBS, the fixed cells were permeabilized with methanol for 10 min at 4 ° C. Next, the washing procedure was repeated and then the cells were incubated with blocking solution (5% BSA in PBS) at room temperature for one hour. The cells were subsequently incubated with diluted-insulin primary antibody (cat. no. ab181547, 1/100 dilution; Abcam, Cambridge, UK) at 4 °C overnight. After being washed with PBS using the same procedure, cells were incubated with FITC or TRITC-conjugated secondary antibodies for one hour at room temperature. Nuclear staining was performed with Hoechst 33342 or DAPI. Immunofluorescence staining was examined using a fluorescence microscope (Eclipse 80i, Nikon, Japan).

### SAMcell-based miRNA functional screening assay

**Figure 1:**
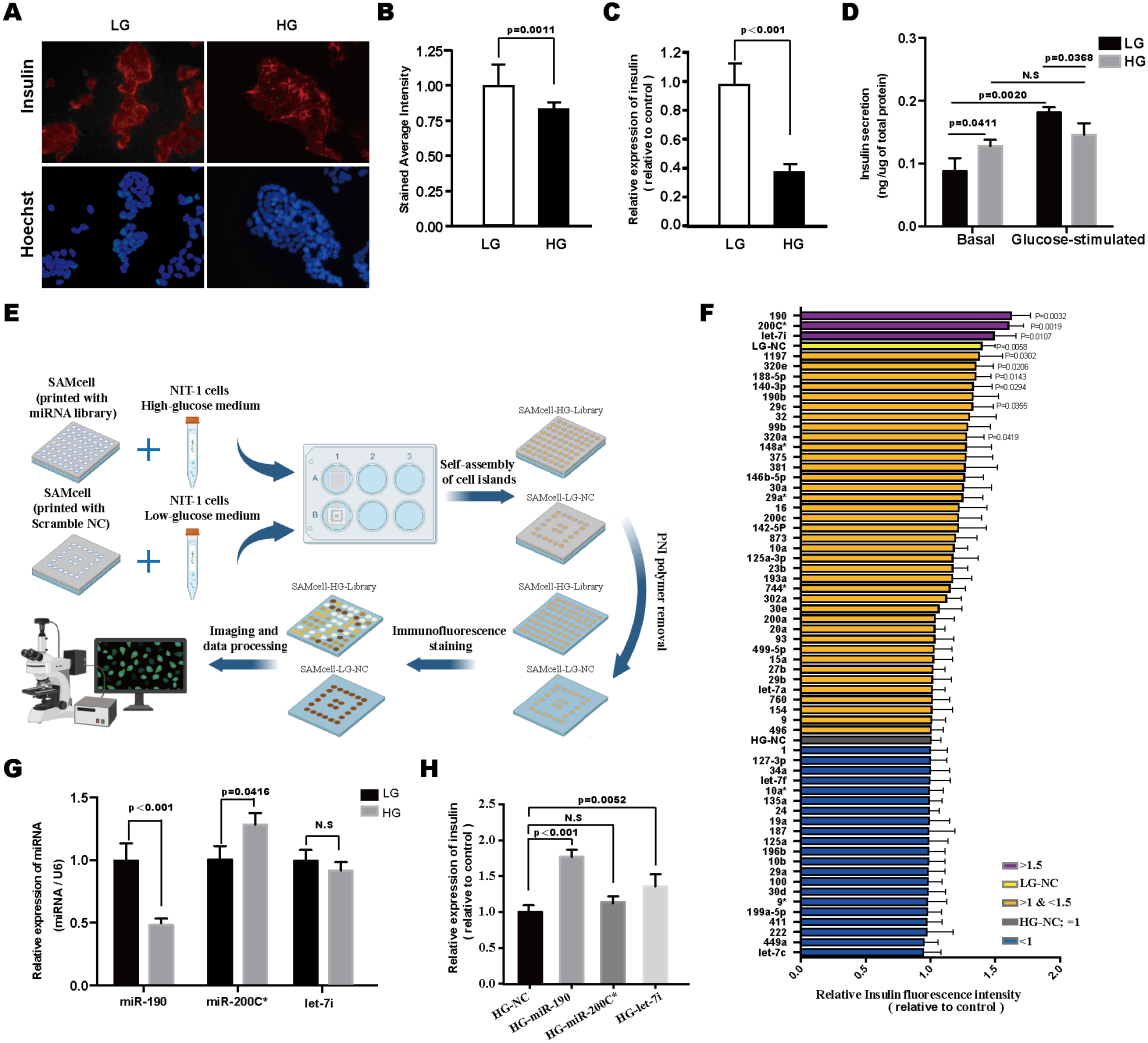
Screening of miRNAs modulating glucotoxicity-induced *β*-cell dysfunction. Representative images (A) and quantification (B) of intracellular insulin content by immunofluorescence staining in NIT-1 cells treated with high-glucose (33.3 mM; HG) for 48 h, NIT-1 cells cultured with low-glucose (5 mM; LG) were used as controls. Cell nuclei were stained using Hoechst 33342. *n* = 5. (C) Insulin mRNA expression was detected by RT-PCR, *n* = 3. (D) Effects of long-term high-glucose exposure on insulin secretion of NIT-1 cells were assessed by GSIS assay, *n* = 3. (E) Schematic diagram of the SAMcell-based miRNA screening assay. (F) The results of the SAMcell-based miRNA screening. High-glucose DMEM (HG; 33.3 mM) cultured NIT-1 cells reverse transfected with the indicated miRNAs, respectively. The horizontal axis represents the relative fluorescence intensity of insulin. Low-glucose (LG; 5 mM) DMEM-cultured NIT-1 cells transfected with scramble NC (LG-NC) were used as the positive control, and high-glucose (HG; 33.3 mM) DMEM cultured NIT-1 cells transfected with scramble NC (HG-NC) were used as the normalization control, *n* = 3. (G) The expression of miR-190, miR-200C* and let-7i was determined by RT-PCR in NIT-1 cells after exposure to high-glucose for 48 h. U6 was used as an internal control, *n* = 3. (H) RT-PCR for insulin mRNA in HG-treated NIT-1 cells after being transfected with the indicated miRNA mimics, *n* = 3. All the quantitative results in this study are presented as the mean ± standard error of the mean with at least three replicate determinations of each data point. Statistical analysis was conducted using the Student’s *t*-test or one-way ANOVA test. *p < 0.05, **p < 0.01, ***p < 0.001.

In the present study, we employed a self-assembled cell microarray (SAMcell)-based functional screening assay to identify miRNAs that could ameliorate glucotoxicity-induced pancreatic *β*-cell dysfunction (Fig. 1F). The fabrication of a SAMcell has been previously described (Zhang et al., 2011). In brief, a layer of poly-N-isopropylacrylamide (PNI) is coated on a glass slide (2.2 cm × 2.2 cm). After drying, the polymer is microfabricated using a shadow mask through oxygen plasma etching. Subsequently, each type of miRNA reverse transfection solution (containing 3 uL 4% Lipofectamine 2000 in Opti-MEM, 1 uL miRNA (100 uM), and 7.25 uL of a 0.2% (w/v) gelatin solution) is printed on the slide according to the polymer pattern. A green fluorescent protein-expressing plasmid is used as a reporter to evaluate the reverse transfection efficiency in NIT-1 cells (Fig. S1). In this assay, a prepared SAMcell was fixed in a six-well plate. Following the cell culture protocol, we added high-glucose medium mixed with NIT-1 cells, and then incubated at 37 ° C with 5% CO_2_.After 48 h of incubation, the plate was moved to room temperature for five minutes and washed with PBS to remove the PNI coated on the SAMcell, which resulted in the formation of individual cell islands. Subsequently, the intracellular insulin level was detected using immunofluorescence staining; nuclear staining was carried out with Hoechst 33342. Finally, the insulin fluorescence intensity was examined and normalized to total cell area using ImageXpress Micro (Molecular Devices, San Jose, CA, USA). Every self-assembled cell island represented cells transfected with the same miRNA. The HG-NC-treated NIT-1 cells (high-glucose cultured; scramble NC transfected) were used as the normalization control, and the relative insulin fluorescence intensity was analyzed.

### Transfection

The miRNA and siRNA used in this study were purchased from GenePharma, inc (Gene Pharma, Beining, China). We used siRNA to target Cybb (si-Cybb: GGUCUUAUUUUGAAGUGUUtt), and a scramble siRNA was used as the negative control. Transfection was performed with Lipofectamine 3000 (Invitrogen, CA).

### Glucose-stimulated insulin secretion (GSIS) in NIT-1 cells

NIT-1 cells were grown in 24-well plates. After 48 h of incubation, cells were rinsed with KRBH buffer, and preequilibrated at 37 ° C in DMEM medium containing 2.5 mM glucose for 5 h. The medium was then changed to KRBH buffer containing 2.5 mM glucose (basal secretion) or KRBH buffer containing 20 mM glucose (glucose-stimulated insulin secretion). The supernatant was collected after 30 min and centrifuged at 2000 g for 3 min. The content of the insulin in the supernatant was measured with an ELISA kit (Abcam, Cambridge, UK) according to the manufacturer’s protocol, and then the NIT-1 cells were lysed and the total amount of cellular protein was analyzed using the BCA assay. Finally, the secreted insulin was normalized with the total protein.

KRBH buffer used in this assay composed of 3.6 mmol/L KCl, 140 mmol/L NaCl, 0.5 mmol/L MgSO_4_, 0.5 mmol/L NaH_2_PO_4_, two mmol/L NaHCO_3_, 1.5 mmol/L CaCl_2_, 10 mmol/L Hepes and 0.1% BSA. The pH was adjusted to 7.4 with NaOH.

### *In vivo* miRNA delivery

We employed a nanoparticle-mediated system for targeted miRNA delivery in vivo. In this approach, mPEG-PCL-g-PDMAEMA (PECgD) was used to conjugate with the miRNA mimics, which can effectively deliver the miRNA to the mouse pancreas (Huang et al., 2012; Lin et al., 2011). The preparation and injection protocol of the PECgD NPs/miRNA complexes was as follows: miRNA mimics (2 µg/ul) were gently mixed with PECgD solution (miRNA/PECgD =1:2, vol./vol.) and the prepared volume of the PECgD NPs/miRNA complex was then calculated to deliver a miRNA dose equivalent to 2 µg/g body weight. After being incubated at room temperature for 20 min, the PECgD NPs/miRNA complexes were administered to the mice *via* tail-vain injection. In this study, we evaluated the *in vivo* delivery efficiency of PECgD NPs/miRNA complexes. The miRNA was labeled with Cy5 dye, and then the PECgD NPs/miRNA-Cy5 complexes were prepared and injected into C57BL/6 mice. Six hours later, mice were euthanized by cervical dislocation, and the fluorescence intensity of the isolated pancreases were examined using the Kodak *in vivo* imaging system (Kodak in Vivo Imaging System FX Pro; Kodak, Rochester, NY, USA). To investigate the function of miR-190 *in vivo*, we randomized 12 db/db (or 12 ob/ob) mice into 2 groups ( *n* = 6 per group), and injected the mice with PECgD NPs/NC or PECgD NPs/miR-190, respectively. Mice with unsuccessful tail vein injections were excluded from the analysis.

### Measurements of fasting blood glucose and serum insulin concentration in mice

After five days of PECgD NPs/miRNA mimics complex injections, food was withheld from the mice for 16 h and fasting blood glucose was measured in blood collected from the tail. The fasting blood glucose was measured three times in each mouse using an automatic glucometer (Johnson & Johnson, New Brunswick, NJ, USA). The concentration of serum insulin was measured with an insulin ELISA kit (Abcam, Cambridge, UK).

### Intraperitoneal glucose tolerance tests (IPGTT)

After five days of PECgD NPs/miRNA mimics complex injections, food was withheld from the mice for 16 h so the mice were in a fasting state. The mice were then injected intraperitoneally with a 20% glucose solution at a volume that was calculated to deliver a glucose dose equivalent to 1 g/kg body weight. Blood samples were taken 15, 30, 60, 90 and 120 min after injection; blood glucose was measured immediately after sampling using an automatic glucometer (Johnson & Johnson, New Brunswick, NJ, USA). At the end of the experiment, mice were then euthanized by cervical dislocation and the pancreases were collected for further analysis.

### RNA isolation and real-time quantitative PCR

Total RNA isolation was carried out using the TRIzol reagent (Tiangen, Beijing, China). A total of 2 ug of each RNA sample was reverse transcribed into cDNA using FastQuant RT Super Mix (Tiangen, Beijing, China). RT-PCR was performed using SYBR Green PCR Master Mix (Tiangen, Beijing, China). A Bugle-Loop miRNA qRT-PCR Kit (RiboBio, Guangzhou, China) was used to quantify miR-190 and U6. The primer sequences used for the RT-PCR were as follows: GAPDH forward, 5′-AGGTCGGTGTGAACGGATTTG- 3′ and reverse, 5′-GGGGTCGTTGATGGCAACA- 3′; Cybb-forward, 5′-ACTCCTTGGGTCAGCACTGG- 3′ and reverse, 5′-GTTCCTGTCCAGTTGTCTTCG- 3′. Insulin forward, 5′-CTTCTACACACCCAAGTCCCG- 3′ and reverse, 5′-GTGCAGCACTGATCCACAATG- 3′.

### Luciferase reporter assay

For the luciferase assays, the WT and mutant Cybb 3′UTR (CTATATC to ACGTGAT; ACATAT to GTCAGC) of Mus musculus were amplified by PCR. The primer sequences for the amplification of Cybb 3′UTR were as follows: Cybb 3′UTR WT-forward, 5′-CTCCGCTCTTTCACCAGGAA- 3′ and reverse, 5′-TAGGGTGCAACACGAAGGTC- 3′. The DNA fragments were cloned into pGL3 plasmid, located at the 3′ end of the firefly luciferase gene. For the luciferase reporter assay, a total of 4 ×10^4^ 293T cells were co-transfected with 200 ng of miRNA mimics, 200 ng of indicated pGL3 firefly luciferase construct and 20 ng of a normalization control pGL3 Renilla luciferase construct. After 48 h, the cells were lysed and the luciferase activity was measured using a Dual-Luciferase Reporter Assay System (Promega, USA) according to the manufacturer’s protocol.

### Protein preparation and western blotting

The NIT-1 cells and mouse pancreases were lysed in a RIPA lysis buffer (50 mM Tris–HCl pH 8.0, 150 mM NaCl, 1% NP-40, 0.5% sodium deoxycholate, and 0.1% SDS) containing 10 mM NaF, 10 mM Na_3_VO_4_, 1 mM PMSF and protease inhibitor cocktail (Roche, USA). The debris was cleared by centrifugation at 12,000 rpm for 20 min at 4 ° C. Total protein concentration was determined with a BCA kit (Beyotime, Jiangsu, China). Then, loaded equal amounts of protein into the wells of a SDS-PAGE gel, ran the gel, and subsequently transferred the protein from the gel to a PVDF membrane (Millipore, Burlington, MA, USA). After transfer, the membrane was rinsed with TBST and incubated in the blocking buffer (5% BSA or 5% non-fat-dried milk in TBST) for 1 h at room temperature; the membrane was incubated in the primary antibodies overnight at 4 ° C at the following dilutions: p38 MAPK (cat. no. 9212; Cell Signaling Technology, Danvers, MA, USA) at 1:1000, phospho-p38 MAPK (cat. no. 9215; Cell Signaling Technology, Danvers, MA, USA) at 1:1000, Akt (cat. no. 9272; Cell Signaling Technology, Danvers, MA, USA) at 1:1000, phospho-Akt (cat. no. 9271; Cell Signaling Technology, Danvers, MA, USA) at 1:1000, gp91phox (sc-130543; Santa Cruz Biotechnology, Dallas, TX, USA) at 1:500, GAPDH (sc-47724; Santa Cruz Biotechnology, Dallas, TX, USA) at 1:1000. After being washed three times with TBST, the membrane was incubated with HRP-conjugated secondary antibody solution (Cell Signaling Technology, Danvers, MA, USA). Following several washes, the band intensity was detected with an ECL system (Transgen, China), and the protein grayscale values were detected and analyzed by the ImageJ software (1.48V; NIH).

### Statistical analysis

All values were represented as means ± SEM. The data was tested for normality with the Komolgorov-Smirnov test. If the data was normally distributed, parametric tests such as the Student’s *t*-test and one-way ANOVA were used. Statistical significance between multiple groups was determined using one-way ANOVA with Tukey’s post hoc test. For comparing two groups, Student’s *t*-test was used. *p* < 0.05 was considered statistically significant.

## Results

### Screening of miRNAs modulating glucotoxicity-induced *β*-cell dysfunction

To simulate the deterioration of *β*-cell function in a diabetic hyperglycemia condition *in vitro*, NIT-1 cells, a mouse pancreatic *β*-cell line, were incubated in the presence of a high concentration of glucose (33.3 mM D-glucose) for 48 h (Yuan et al., 2010a). Immunofluorescence staining and RT-PCR analysis revealed that long-term exposure of NIT-1 cells to high-glucose (HG) conditions significantly decreased intracellular insulin content and inhibited insulin gene expression (Figs. 1A–1C). Subsequently, insulin secretion was evaluated through the glucose-stimulated insulin secretion (GSIS) assay. The results showed increased basal insulin secretion (2.5 mmol/L D-glucose in KRBH buffer) and decreased GSIS (20 mmol/L D-glucose in KRBH buffer) in HG pre-treated NIT-1 cells (Fig. 1D). These results demonstrated that long-term exposure of NIT-1 cells to HG conditions caused *β*-cell glucotoxicity, which was accompanied by impaired insulin biosynthesis and secretion.

To identify the miRNAs whose modulation in pancreatic *β*-cells is capable of reversing glucotoxicity-induced *β*-cell dysfunction, we performed a SAMcell-based miRNA functional screening in NIT-1 cells using a library of 62 conserved miRNAs (Fig. 1E). The screening identified three miRNAs (miR-let-7i, miR-200C* and miR-190) that increased intracellular insulin content in HG-treated NIT-1 cells more than 1.5-fold compared to the HG-NC group (Fig. 1F). Of the three selected miRNAs, miR-190 was downregulated in the NIT-1 cells under glucotoxic conditions (Fig. 1G), and the overexpression of miR-190 reversed the glucotoxicity-induced repression of insulin expression (Fig. 1H).

### Overexpression of miR-190 exerts protective effects against glucotoxicity-induced NIT-1 cell damage

**Figure 2:**
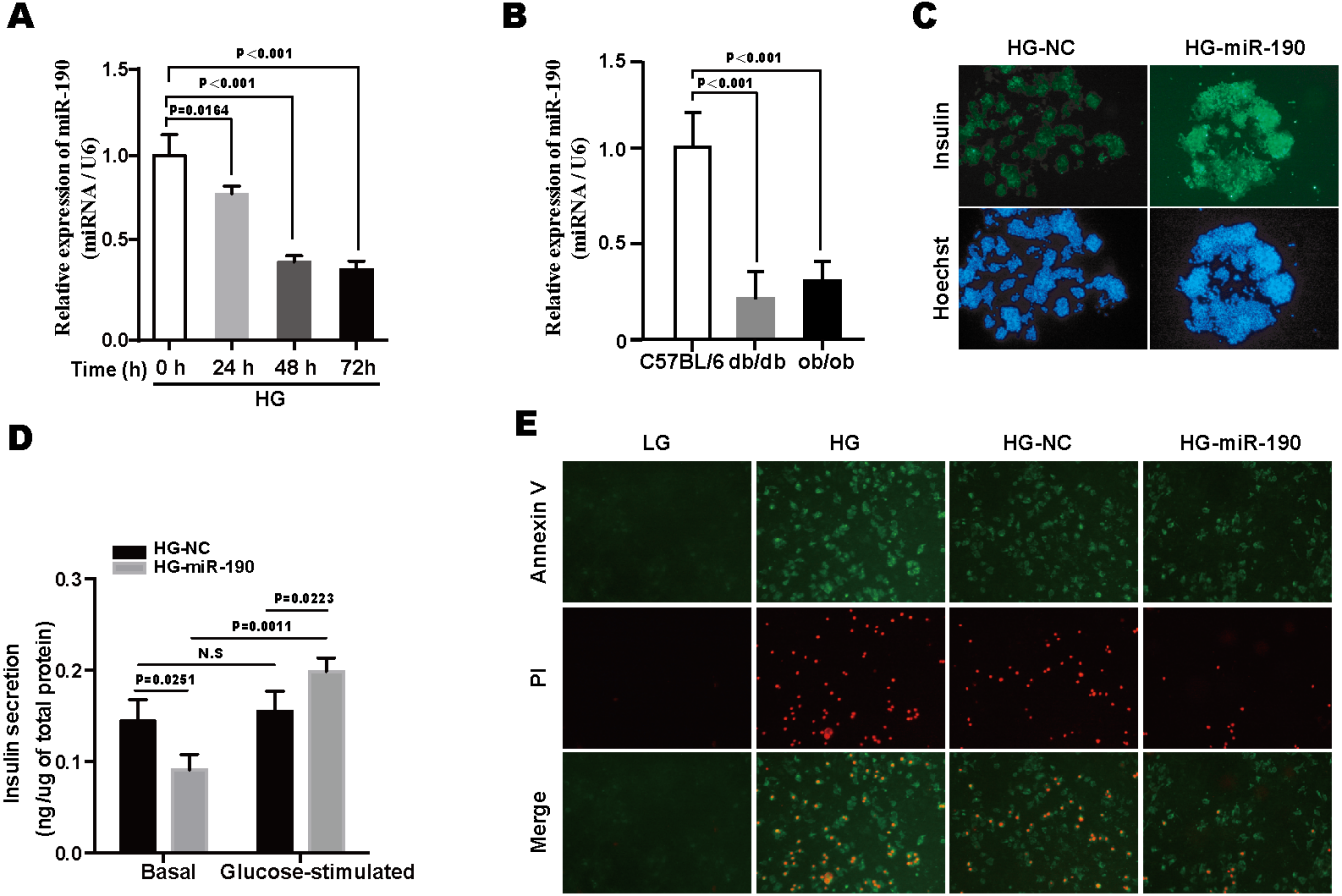
Overexpression of miR-190 exerts protective effects against glucotoxicity-induced NIT-1 cell damage. (A) miR-190 expression was determined by RT-PCR in NIT-1 cells after exposure to high-glucose for 0, 24, 48 and 72 h, respectively, U6 was used as an internal control, *n* = 3. (B) The expression of miR-190 was detected by RT-PCR in the pancreas of *db/db* and *ob/ob* mice, C57BL/6 mice were used as control, *n* = 3 per group. (C) Immunofluorescence staining was used to detect the intracellular insulin content of NIT-1 cells, cell nuclei were stained by Hoechst 33342. (D) Effects of miR-190 transfection on insulin secretion was determined by GSIS assay in HG-treated NIT-1 cells, *n* = 3. (E) Representative images of Annexin V/PI staining. All the quantitative results in this study are presented as the mean ± standard error of the mean with at least three replicate determinations of each data point. Statistical analysis was conducted using the Student’s *t*-test or one-way ANOVA test. *p < 0.05, **p < 0.01, ***p < 0.001.

We next focused on miR-190. As shown in the Fig. 2A, miR-190 expression was decreased in a time-dependent manner as NIT-1 cells were exposed to high-glucose (Fig. 2A). Moreover, the downregulation of miR-190 was detected in the pancreas of *db/db* and *ob/ob* mice (Fig. 2B). These findings indicate that miR-190 might be involved in glucotoxicity-induced pancreatic *β*-cell failure. We further validated the effects of miR-190 and the results showed that consistent with the data of the SAMcell-based screening assay, miR-190 significantly upregulated intracellular insulin content in HG-treated NIT-1 cells (Fig. 2C). In order to determine the effects of miR-190 on cell survival and insulin-secretion function in NIT-1 cells, Annexin V/PI staining and a GSIS assay were performed, respectively. The results revealed that the overexpression of miR-190 improved defective GSIS and inhibited apoptosis in HG-treated NIT-1 cells (Figs. 2D–2E).

### miR-190 attenuates the over-production of ROS and alleviates oxidative stress

Glucotoxicity-induced *β*-cell failure has been attributed, at least partly, to the excessive production of ROS (Eguchi et al., 2021; Yuan et al., 2010a). Previous studies have indicated that ROS accumulation could result in oxidative stress, which is a deleterious process that can alter major pathways important for *β*-cell function and survival (Eguchi et al., 2021). It has been confirmed that PI3K/Akt signaling is a critical regulator in *β*-cell function and survival, and inactivation of this pathway occurs in response to oxidative stress (Elghazi & Bernal-Mizrachi, 2009; Yuan et al., 2010a). Another important signaling pathway activated by oxidative stress in *β*-cells is the p38 MAPK pathway; the activation of p38 MAPK has been implicated in *β*-cell apoptosis (Yuan et al., 2010a; Yuan et al., 2010b).

**Figure 3:**
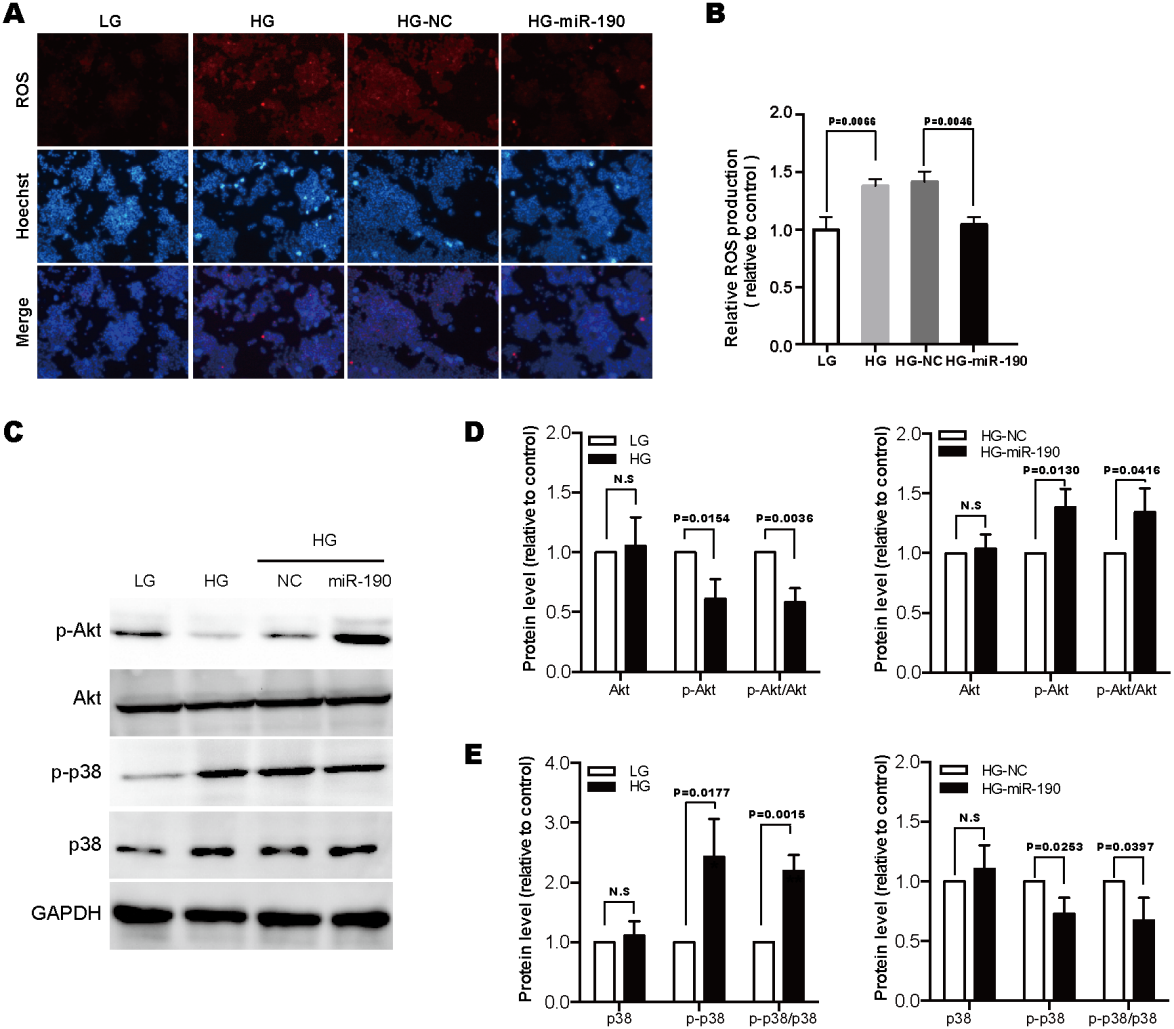
MiR-190 attenuates the over-production of ROS and alleviates oxidative stress. Representative images (A) and quantification (B) of DHE staining in NIT-1 cells under the indicated conditions, *n* = 3. (C) Western blot was used to detect the expression levels of Akt, p38 and their phosphorylated proteins. (D) and (E) Protein grayscale values were detected and analyzed by ImageJ software, *n* = 3. All the quantitative results in this study are presented as the mean ± standard error of the mean with at least three replicate determinations of each data point. Statistical analysis was conducted using the Student’s *t*-test or one-way ANOVA test. *p < 0.05, **p < 0.01, ***p < 0.001.

In the present study, we found that HG-stimulation promoted ROS over-production in NIT-1 cells, but these effects were counteracted by the overexpression of miR-190 (Figs. 3A and 3B). Next, to determine whether oxidative stress-associated pathways, PI3K/Akt and p38 MAPK, were involved in miR-190-mediated *β*-cell protection, we transfected HG-treated NIT-1 cells with miR-190 and evaluated the active state of these two pathways by western blot. As shown in Figs. 3C–3E, we found that prolonged HG exposure downregulated the phosphorylation of Akt, but increased p38 phosphorylation. Importantly, the overexpression of miR-190 dramatically eliminated the high-glucose-induced alterations of these pathways. Based on the above results, we conclude that miR-190 attenuates glucotoxicity-induced ROS elevation, and has protective effects against oxidative stress through modulating the PI3K/Akt and p38 MAPK pathways.

### Cybb is a direct target gene of miR-190

**Figure 4:**
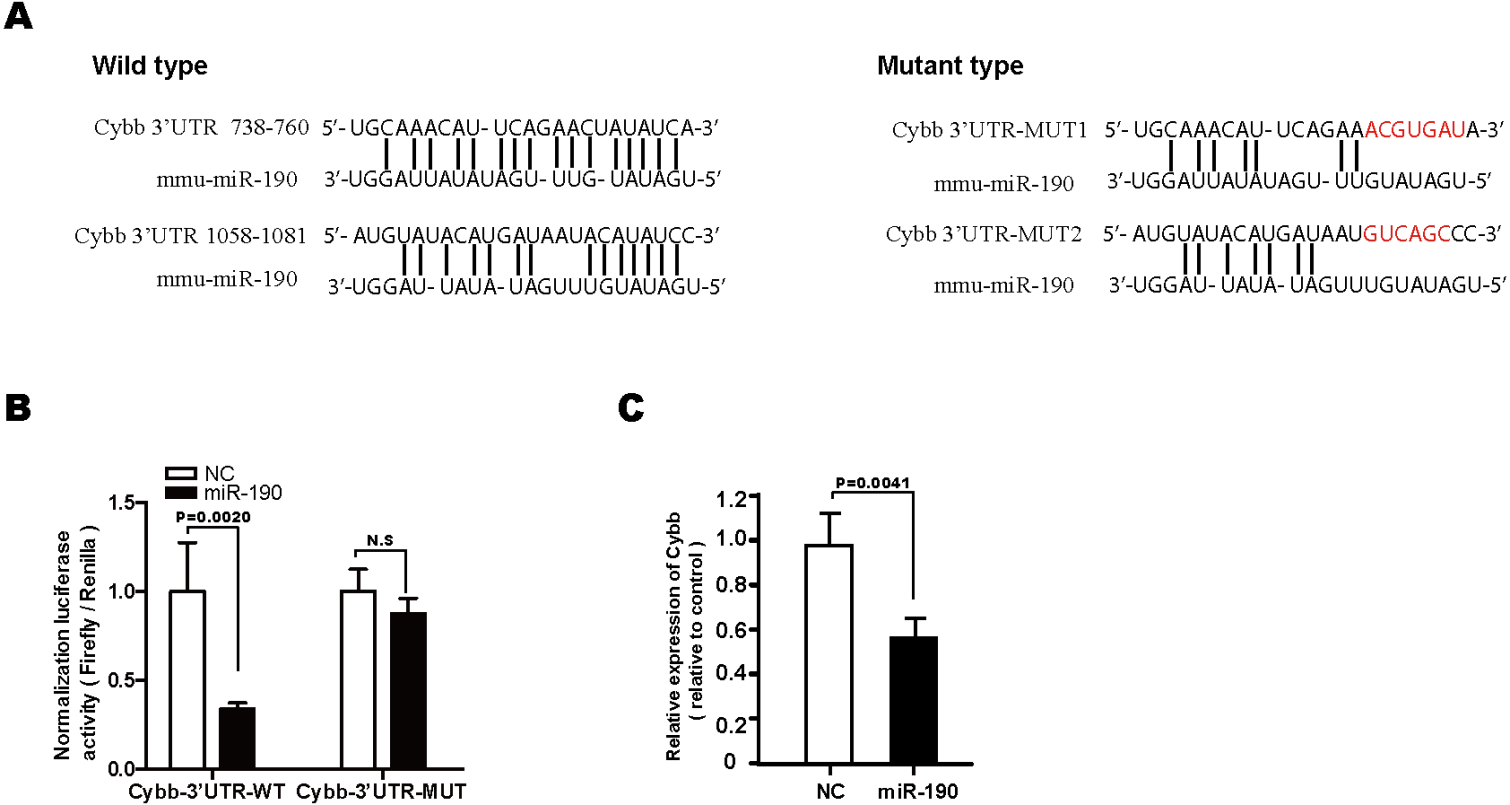
Cybb is a direct target gene of miR-190. (A) Sequence alignment of miR-190 with potential binding site with wild-type (WT) and mutant (MUT) Cybb 3′UTR. (B) miR-190 mimics or scramble NC were co-transfected with luciferase reporter constructs containing WT or MUT Cybb 3′UTR in 293T cells, and relative luciferase activity were measured, *n* = 4. (C) RT-PCR analysis was performed to detect the expression level of Cybb in NIT-1 cells transfected with miR-190 mimics. Scrambe NC mimics were used as the control, *n* = 3. All the quantitative results in this study are presented as the mean ± standard error of the mean with at least three replicate determinations of each data point. Statistical analysis was conducted using the Student’s *t*-test or one-way ANOVA test. **p < 0.01.

We further investigated the molecular mechanisms underlying the protective effects of miR-190 against glucotoxicity-induced *β*-cell damage and found that Cybb, which encodes the gp91phox protein, was predicated to be a key target gene of miR-190 by miRanda (Fig. 4A). The gp91phox protein is the catalytic subunit of the NOX2 complex, which plays a critical role in the oxidative damage of pancreatic *β*-cells (Kowluru & Kowluru, 2014; Yuan et al., 2010a). To confirm the binding activation of Cybb 3′UTR with miR-190, luciferase reporter plasmids with the wild-type (WT) or the mutant (MUT) Cybb 3′UTR were constructed and co-transfected with miR-190 mimics in 293T cells. The results demonstrated that miR-190 reduced luciferase activity in pGL3-Cybb- 3′UTR-WT transfected cells (Fig. 4B). Moreover, the overexpression of miR-190 markedly decreased Cybb expression levels in NIT-1 cells (Fig. 4C). These data demonstrated that miR-190 could negatively regulate the expression of Cybb by directly binding to its 3′UTR.

### Knockdown of Cybb can mimic the *β*-cell-protecting effects of miR-190

**Figure 5:**
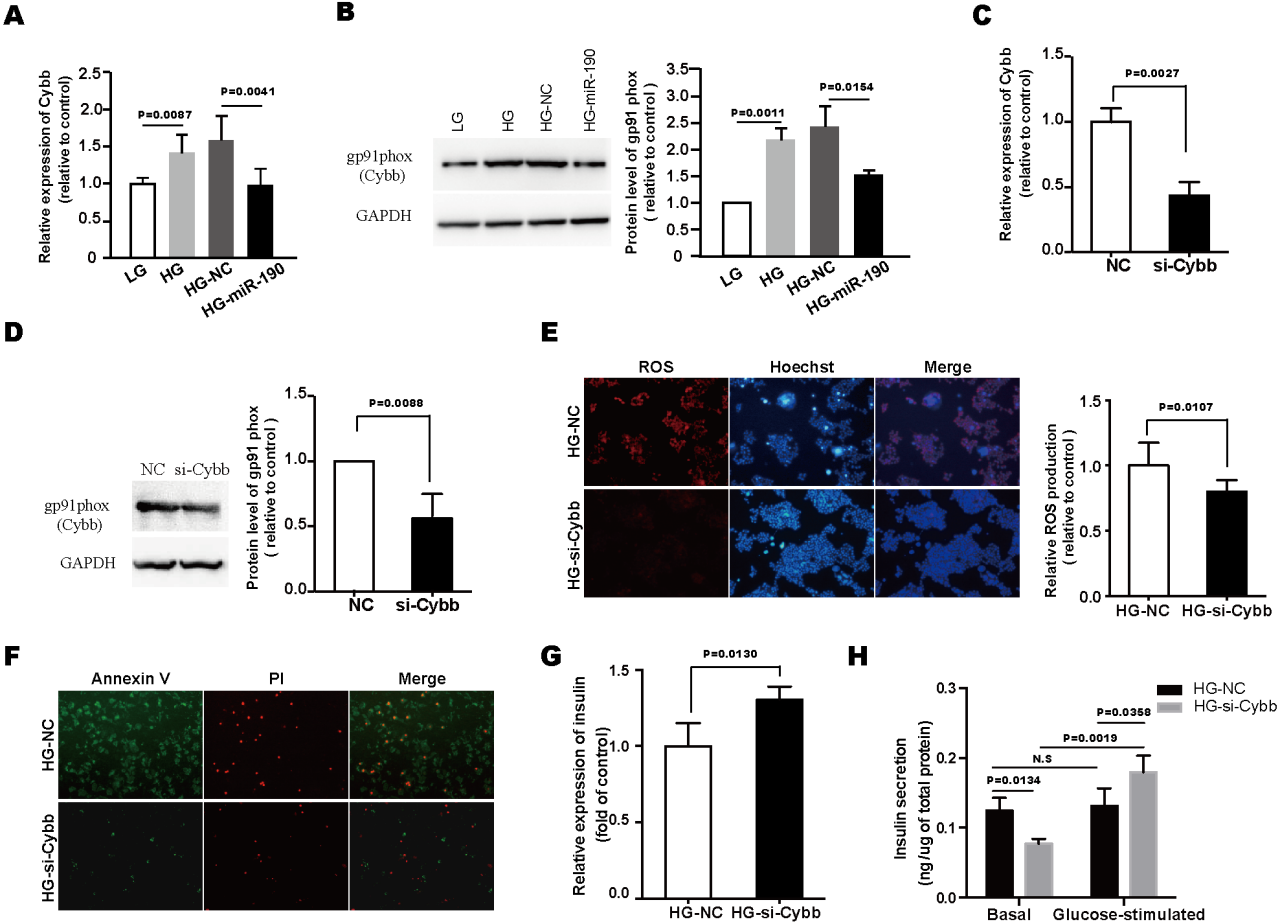
Knockdown of Cybb can mimic the *β*-cell-protecting effects of miR-190. (A) The expression level of Cybb in NIT-1 cells was determined by RT-PCR, *n* = 6. (B) Cybb expression level in NIT-1 cells was detected by western blot, GAPDH was used as the loading control, *n* = 3. (C) and (D) Verification of knockdown of Cybb in NIT-1 cells by RT-PCR (C) and western blot (D), Scramble NC mimics were used as controls, *n* = 3. (E) ROS production was detected by DHE staining, *n* = 8. (F) Cell apoptosis was evaluated by AnnexinV/PI staining. (G) The expression level of insulin in NIT-1 cells was detected by RT-PCR, *n* = 4. (H) Effects of Cybb knockdown on insulin secretion of NIT-1 cells were assessed by GSIS assay, *n* = 3. All the quantitative results in this study are presented as the mean ± standard error of the mean with at least three replicate determinations of each data point. Statistical analysis was conducted using the Student’s *t*-test or one-way ANOVA test. *p < 0.05, **p < 0.01, ***p < 0.001.

We next examined the expression levels of Cybb in HG-stimulated NIT-1 cells, and found that prolonged high-glucose exposure significantly upregulated the expression of Cybb (Figs. 5A and 5B). However, this alteration was reversed by the overexpression of miR-190 (Figs. 5A and 5B). To further clarify the role of Cybb in miR-190-mediated anti-glucotoxicity effects, Cybb expression was knocked down using siRNA (Figs. 5C and 5D). The results of DHE staining showed that si-Cybb significantly inhibited the excessive production of ROS induced by high-glucose (Fig. 5E). Furthermore, Annexin V/PI staining revealed that the glucotoxicity-induced apoptosis of NIT-1 cells was inhibited by the knockdown of Cybb (Fig. 5F). Moreover, we assessed the role of Cybb on regulating *β*-cell function. The results indicated that si-Cybb increased the downregulated insulin biogenesis and improved insulin secretion in HG-treated NIT-1 cells (Figs. 5G and 5H). Taken together, these findings suggest that miR-190 protected NIT-1 cells against glucotoxicity by negatively regulating the expression of Cybb.

### *In vivo* studies of miR-190 mediated anti-diabetic effects in db/db and ob/ob mice

**Figure 6:**
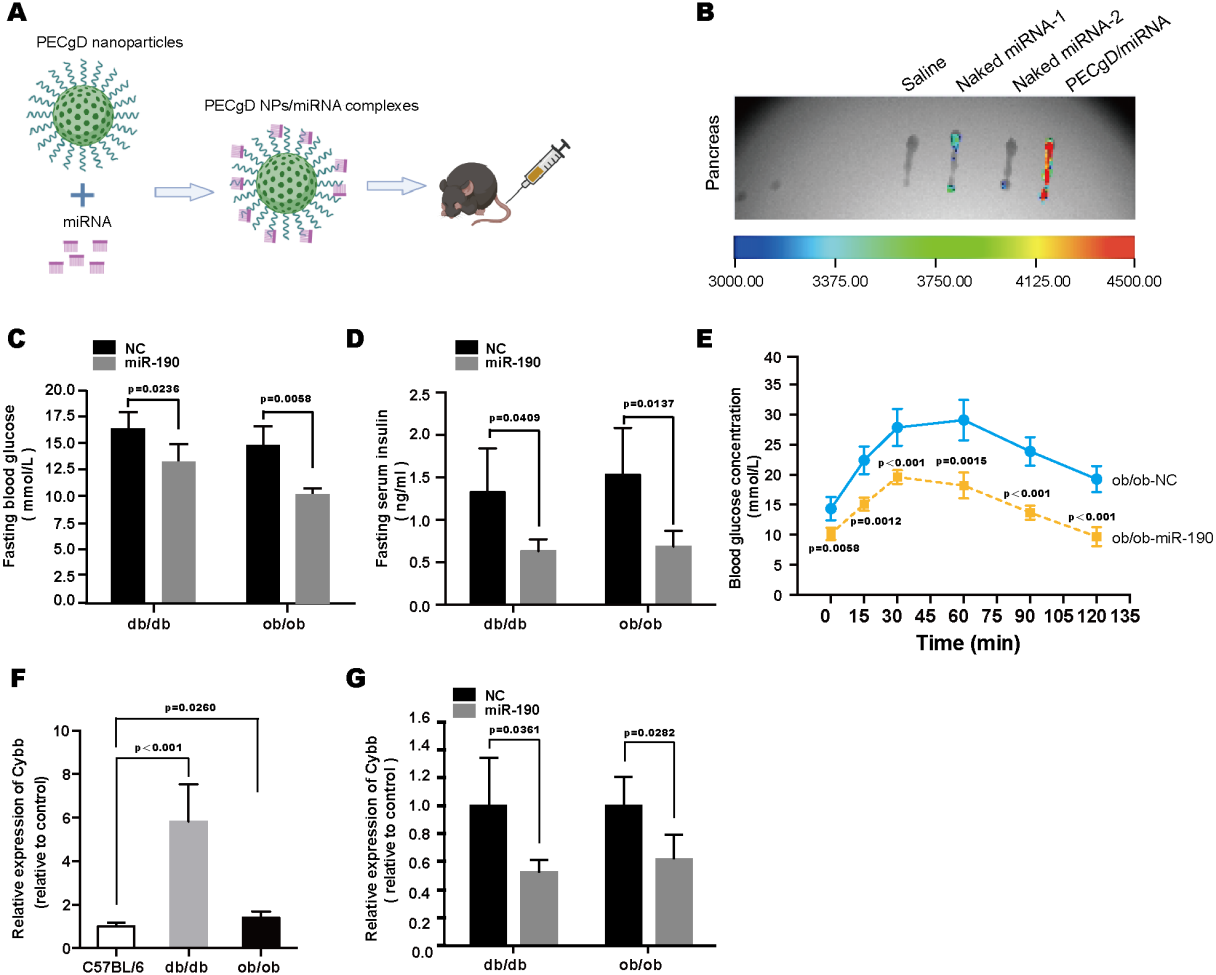
*In vivo* studies of miR-190 mediated anti-diabetic effects in *db/db* and *ob/ob* mice. (A) Schematic illustration of PECgD NPs/miRNA complexes preparation and *in vivo* injection. (B) Fluorescence detection of isolated pancreas from C57BL/6 mice after administration *via* tail-vein injection, including the saline group (*n* = 1), the naked miRNA group (*n* = 2), and the PECgD/miRNA group (*n* = 1). (C) and (D) Fasting blood glucose (C) and fasting serum insulin (D) were detected in PECgD NPs/miR-190-injected diabetic mice (*db/db* and *ob/ob*), and mice injected with PECgD NPs/NC were used as controls, *n* = 4 per group. (E) Glucose tolerance of PECgD NPs/miR-190-injected *ob/ob* mice was determined by IPGTT, and mice injected with PECgD NPs/NC were used as controls, *n* = 4 per group. (F) RT-PCR was performed to detect the expression level of Cybb in the pancreas of *db/db* and *ob/ob* mice, and C57BL/6 mice were used as controls, *n* = 5 per group. (G) RT-PCR was performed to detect the expression level of Cybb in the pancreas of PECgD NPs/miR-190-injected diabetic mice (*db/db* and *ob/ob*), and mice injected with PECgD NPs/NC were used as controls, *n* = 4 per group. All the quantitative results in this study are presented as the mean ±standard error of the mean with at least three replicate determinations of each data point. Statistical analysis was conducted using the Student’s *t*-test or one-way ANOVA test. *p < 0.05, **p < 0.01, ***p < 0.001.

To explore the function of miR-190 *in vivo*, we employed the PECgD nanoparticle for the targeted delivery of miR-190 mimics to the pancreas (Huang et al., 2012). The preparation and injection of the PECgD NPs/miRNA complex was described in the methods section (Fig. 6A). We evaluated miRNA delivery efficiency. As shown in Fig. 6B, compared with the naked miRNA group, the PECgD NPs/miRNA complexes showed relatively stronger fluorescence signals, which means the PECgD NPs/miRNA system can efficiently deliver miRNA to the pancreas *in vivo*. Then, to clarify the anti-diabetic effects of PECgD NPs/miR-190 complexes, *db/db* and *ob/ob* mice were intravenously injected with PECgD NPs/NC or PECgD NPs/miR-190 complexes (dose: 2 µg/g). Five days after injection, the concentrations of fasting blood glucose and fasting serum insulin were measured. Without exception, all subjects survived until the end of the experiment, with no signs of illness such as increased temperature, fur changes, or trembling. The results showed that in both *db/db* and *ob/ob* mice, the PECgD NPs/miR-190 group presented lower concentrations of fasting blood glucose and fasting serum insulin, compared with the PECgD NPs/NC group (Figs. 6C and 6D). In addition, we detected the effects of PECgD NPs/miR-190 on glucose tolerance, and the results of the IPGTT revealed an improvement in glucose tolerance in the PECgD NPs/miR-190 injected *ob/ob* mice (Fig. 6E). Furthermore, to verify the regulation of Cybb expression by miR-190 *in vivo*, we examined the expression level of Cybb in the pancreas. As shown in Figs. 6F and 6G, the Cybb expression level in the pancreas of diabetic mice (*db/db* and *ob/ob*) was significantly up-regulated (Fig. 6F), while PECgD NPs/miR-190 injection dramatically decreased Cybb expression levels (Fig. 6G). Based on these results, we conclude that the targeted delivery of miR-190 mimics to the pancreas could alleviate diabetes symptoms.

## Discussion

MicroRNAs, or miRNAs are short, non-coding RNAs that modulate gene expression in a post-transcriptional manner. They are involved in almost every physiological and pathological process (Krol, Loedige & Filipowicz, 2010). Many miRNAs have been identified as diagnostic biomarkers and potential therapeutic targets for various diseases, including diabetes (Kumar et al., 2012; Mendell & Olson, 2012). Most of the previous research done of miRNA in diabetes has focused on the differential expression profile of miRNAs in a diabetic model. In the present study, we designed a SAMcell-based functional screening assay to identify miRNAs that are capable of regulating the dysfunction of *β*-cells induced by glucotoxicity and thus might be potential targets for novel therapeutic approaches to diabetes. In this study, we found that miR-190 could effectively restore insulin biogenesis and secretion in NIT-1 cells under high-glucose stimulation. miR-190 is a conserved miRNA between human and mice, the miR-190 encoding gene is located in the intronic regions of the Tln2 protein coding gene on chromosome 9 in mice and chromosome 15 in humans. The role of miR-190 has been investigated in various studies. It has been reported that the abnormal expression of miR-190 is involved in several diseases, including diabetes mellitus and its complications (Yu & Cao, 2019). Mirra P et al. found that the downregulation of miR-190 plays an important role in methyglyoxal-induced endothelial insulin resistance by increasing KRAS (Mirra et al., 2017). In another study, Yang et al. demonstrated that miR-190 is downregulated in the lumbar dorsal horn of diabetic mice, and that the up-regulation of miR-190 could significantly weaken diabetic neuropathic pain by targeting SLC17A6 (Yang et al., 2017). However, the functions of miR-190 in diabetes mellitus are not yet fully understood. Here, our findings first expose the protective role of miR-190 in glucotoxicity-induced *β*-cell failure.

Further mechanistic studies demonstrated that Cybb was the direct target gene of miR-190. Cybb encodes the gp91phox protein, which is the catalytic subunit of the NOX2 complex (Bedard & Krause, 2007). Recent studies have demonstrated that NOX2 is a major source of extra mitochondrial superoxide in pancreatic *β*-cells (Newsholme et al., 2009). Increased activity of NOX2 leads to the excessive generation of ROS, which subsequently results in oxidative stress damage in *β*-cells (Eguchi et al., 2021). In the present study, Cybb was identified to be a direct target gene of miR-190. It was found to be up-regulated in the pancreas of diabetic mice and HG-treated NIT-1 cells. The overexpression of miR-190 suppressed the expression of Cybb, thereby alleviating oxidative stress and eliminating the high-glucose induced alterations of the PI3K/Akt and p38 MAPK pathways. Both miR-190 overexpression and Cybb knockdown could attenuate glucotoxicity-induced apoptosis and dysfunction of NIT-1 cells. Thus, our data demonstrate that the miR-190/Cybb axis plays a critical role in glucotoxicity-induced *β*-cell damage.

MicroRNAs are potential therapeutic agents for the treatment of human diseases. One major challenge to miRNA-based therapy, however, is the efficient and precise delivery of miRNA to targeted sites *in vivo* (Chen, Gao & Huang, 2015). In this study, in order to deliver the miR-190 mimics to the pancreas, we employed the PECgD nanoparticle-based miRNA delivery system (Huang et al., 2012; Lin et al., 2011). Our results confirmed that miRNA mimics accumulate in the mouse pancreas, and that the injection of PECgD NPs/miR-190 complex into the diabetic mice could down-regulate the Cybb expression in the pancreas and ameliorate diabetic symptoms. It has also been reported that PECgD NPs could deliver siRNA/miRNA to the liver (Lin et al., 2011), while the liver is one of the main target organs for insulin, whether the PECgD NPs/miR-190 complex could affect glucose-metabolism partially through the liver requires further investigation.

## Conclusions

In conclusion, this study is the first to demonstrate that miR-190 attenuates NOX2-mediated ROS generation by negatively regulating the expression of Cybb, thereby protecting *β*-cells against glucotoxicity. The PECgD NPs-mediated *in vivo* delivery of miR-190 could ameliorate diabetic symptoms. These results indicate that our work might provide a new therapeutic target for the prevention and treatment of T2DM.

##  Supplemental Information

10.7717/peerj.13849/supp-1 Supplemental Information 1 The original images of western blots for Fig. 3C

10.7717/peerj.13849/supp-2 Supplemental Information 2 The original images of western blots for Fig. 5B

10.7717/peerj.13849/supp-3 Supplemental Information 3 The original images of western blots for Fig. 5D

10.7717/peerj.13849/supp-4 Figure S1 The reverse transfection efficiency of NIT-1 cells A green fluorescent protein-expressing plasmid (pcDNA3.1-GFP) was used as a reporter to evaluate the reverse transfection efficiency of NIT-1 cells.

10.7717/peerj.13849/supp-5 Data S1 Raw data The raw data file can be opened in Excel.

10.7717/peerj.13849/supp-6 Supplemental Information 6 Author Checklist

## Additional Information and Declarations

### Data Availability

The following information was supplied regarding data availability: The raw measurements are available in the Supplemental Files.
