# Supplementary figures and images for: MiR-190 ameliorates glucotoxicity-induced dysfunction and apoptosis of pancreatic β-cells by inhibiting NOX2-mediated reactive oxygen species production
Source: PeerJ. 2022 Aug 10;10:e13849. doi: 10.7717/peerj.13849 (PMC9375543; doi:10.7717/peerj.13849)

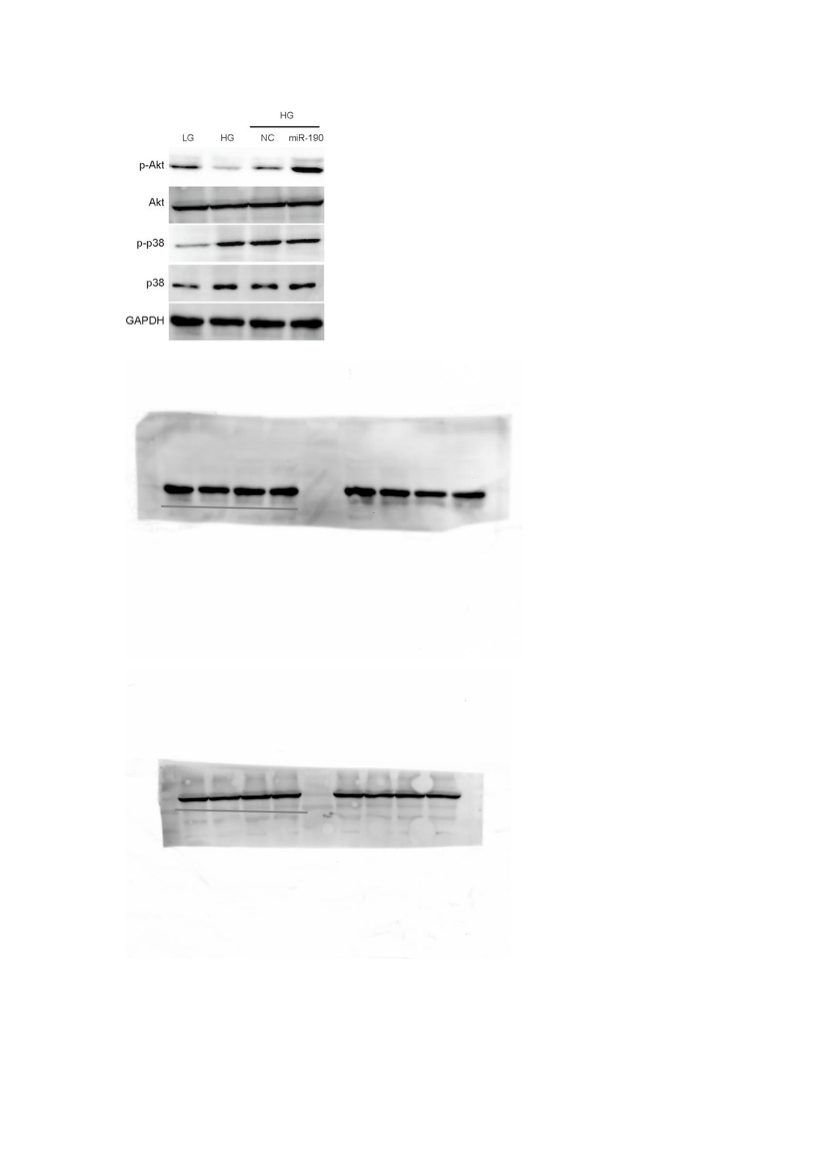

Supplement: Supplemental Information 1 [file peerj-10-13849-s001.png]

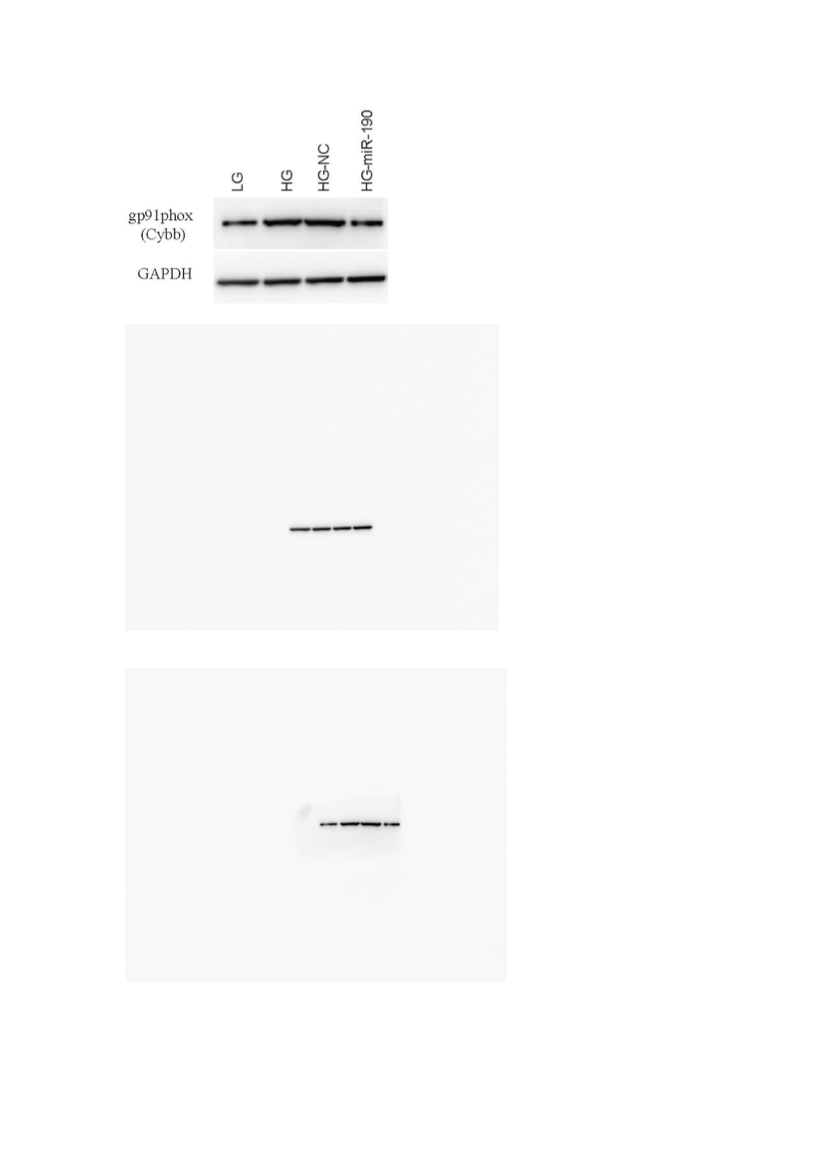

Supplement: Supplemental Information 2 [file peerj-10-13849-s002.png]

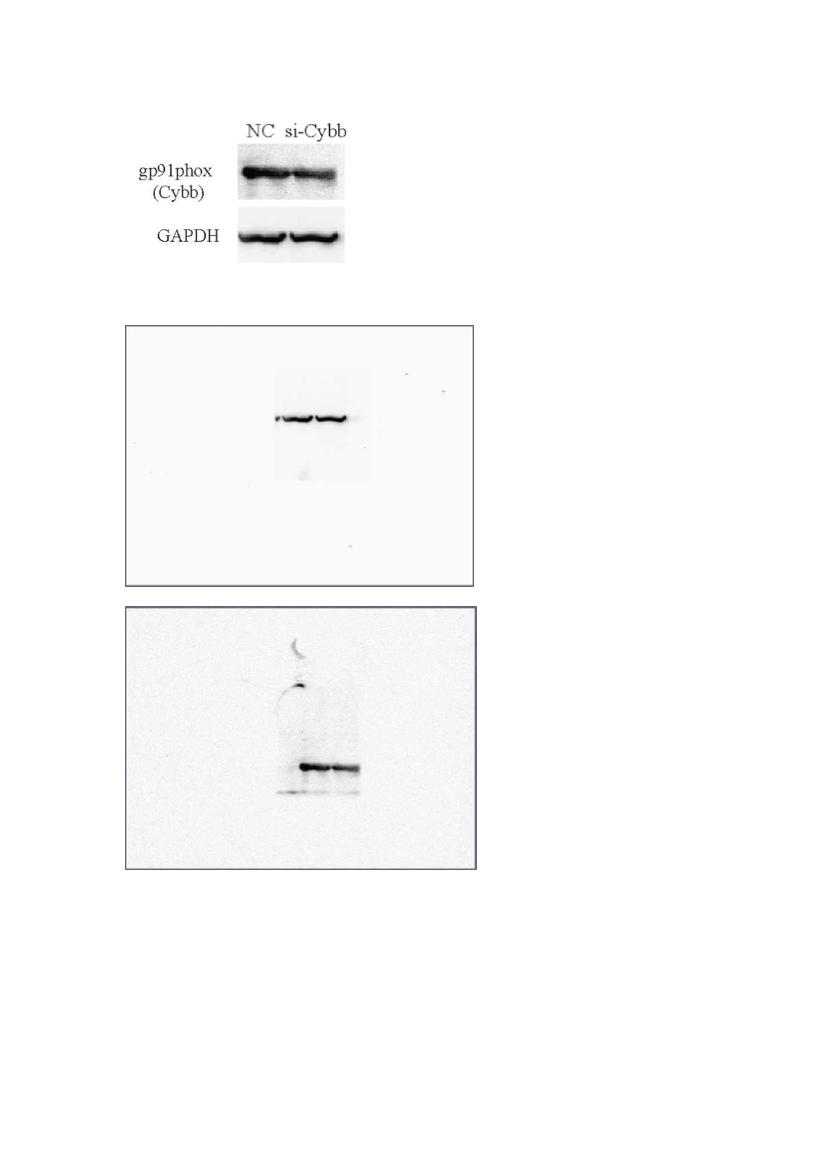

Supplement: Supplemental Information 3 [file peerj-10-13849-s003.png]

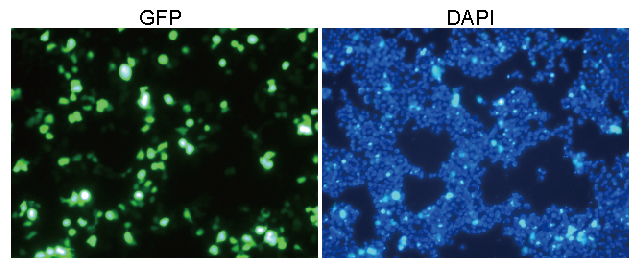

Supplement: Figure S1 — A green fluorescent protein-expressing plasmid (pcDNA3.1-GFP) was used as a reporter to evaluate the reverse transfection efficiency of NIT-1 cells. [file peerj-10-13849-s004.png]
